# Reactive intermediates in naquotinib metabolism identified by liquid chromatography-tandem mass spectrometry: phase I metabolic profiling

**DOI:** 10.1039/c9ra00224c

**Published:** 2019-04-01

**Authors:** Mohamed W. Attwa, Adnan A. Kadi, Haitham AlRabiah, Hany W. Darwish

**Affiliations:** Department of Pharmaceutical Chemistry, College of Pharmacy, King Saud University P. O. Box 2457 Riyadh 11451 Kingdom of Saudi Arabia mzeidan@ksu.edu.sa akadi@ksu.edu.sa halrabiah@ksu.edu.sa hdawish@ksu.edu.sa +966 1146 76 220 +966 1146 77343; Students' University Hospital, Mansoura University Mansoura 35516 Egypt; Analytical Chemistry Department, Faculty of Pharmacy, Cairo University Kasr El-Aini St. Cairo 11562 Egypt

## Abstract

Tyrosine kinase inhibitors (TKIs) are very efficient for the treatment of EGFR-mutated lung cancer and show improved therapeutic efficacy. However, treatment with both first- and second-generation TKIs results in acquired resistance and is related to various toxicities; the EGFR T790M mutation has been associated with this resistance. Naquotinib (ASP8273, NQT) is a novel third-generation epidermal growth factor receptor tyrosine kinase inhibitor that has been shown to be more potent than osimertinib in the management of L858R plus T790M mutations. However, its bioactivation may occur and promote the formation of reactive electrophiles that are toxic. We hypothesize that these reactive intermediates are potentially involved in the side effects of NQT. Reactive metabolites are often formed by phase I metabolic reactions and cannot be characterized directly as they are transient in nature. Using liquid chromatography-tandem mass spectrometry (LC-MS/MS), we screened for *in vitro* metabolites of NQT formed during incubation with human liver microsomes and evaluated the generation of reactive electrophiles using capturing agents, such as methoxyamine and potassium cyanide, as nucleophiles that form stable adducts for identification by LC-MS/MS. Eight NQT phase I metabolites were found that had been formed by *N*-demethylation, oxidation, hydroxylation, and reduction. In addition, three reactive electrophiles, two aldehydes, and one iminium ion were identified, and the corresponding bioactivation mechanisms were proposed. The reported side effects of NQT may be related to the generation of reactive metabolites. Based on a literature review, this may be the first study of *in vitro* phase I metabolites, detailed structural characterizations, and NQT reactive intermediates.

## Abstract 2

LC-MS/MS was used to screen for *in vitro* metabolites of NQT formed during incubation with human liver microsomes (HLMs) and then evaluated the generation of reactive electrophiles using capturing agents.

## Graphical abstract

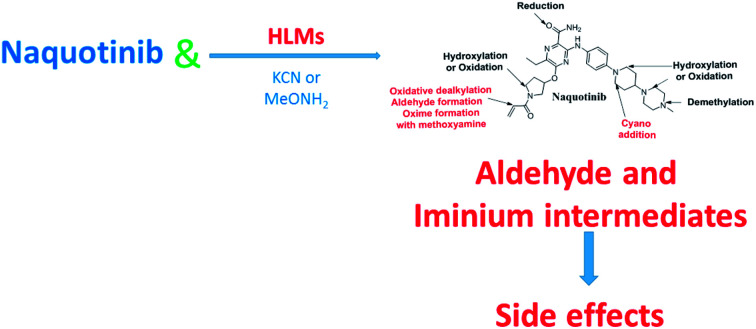

## 1. Introduction

Non-small cell lung cancer (NSCLC) is a common lung cancer subgroup,^1–5^ accounting for approximately 90% of all cases of lung cancer. Epidermal growth factor receptor (EGFR) signaling pathway was recently identified as an important potential NSCLC therapeutic target.^6^ Tyrosine kinase inhibitors (TKIs), which regulate the EGFR, are very efficient for the treatment of EGFR-mutated lung cancer and show improved therapeutic efficacy. First-line TKIs regulating EGFR (*e.g.*, gefitinib and erlotinib) show good initial responses against tumors harboring these EGFR mutations.^7,8^ However, treatment with these inhibitors results in acquired resistance in 60% of patients and is related to various toxicities,^9,10^ thereby reducing their therapeutic efficacies.^11,12^ Accordingly, second-generation irreversible EGFR TKIs (*e.g.*, avitinib and dacomitinib) have also been developed.^13,14^ Unfortunately, NSCLCs have been shown to exhibit resistance to both first- and second-generation EGFR-TKIs within the first year of treatment.^15^ Notably, the EGFR T790M mutation has been identified in approximately 50% of NSCLC cases showing resistance to first- and second-generation EGFR-TKIs.^8,13^

Third-generation TKIs have maintained the benefits of second-generation drugs by blocking mutant EGFR and overcoming resistance induced by the T790M mutation.^13,14^ Naquotinib (ASP8273, NQT) is a novel third-generation EGFR-TKI that was found to be more potent than osimertinib against T790M plus L858R mutations (T790M + L858R). Moreover, NQT and osimertinib have been shown to have a wide therapeutic window and comparable efficacy for cells with EGFR exon 20 insertions.^16^

Metabolic detoxification involves pathways that transform xenobiotics and endogenous compounds into more hydrophilic molecular species to facilitate excretion outside the human body. The generated metabolites are usually less toxic than the parent molecules. However, in rare cases, bioactivation may occur and promote the formation of reactive electrophiles that are more toxic.^17–19^ Reactive electrophiles are electron deficient and can modify DNA and proteins by establishing covalent bonds that are considered the first step in drug-mediated organ toxicities.^20,21^ Monitoring of reactive metabolite production is a critical task in studying drug-induced toxicity. Reactive metabolites are often formed by phase I metabolic reactions and cannot be characterized directly as they are transient in nature. Instead, a capturing nucleophile can be used for reactive intermediates to trap the adducts formed, which are stable and can be characterized by liquid chromatography-tandem mass spectrometry (LC-MS/MS).^22,23^

**Fig. 1:**
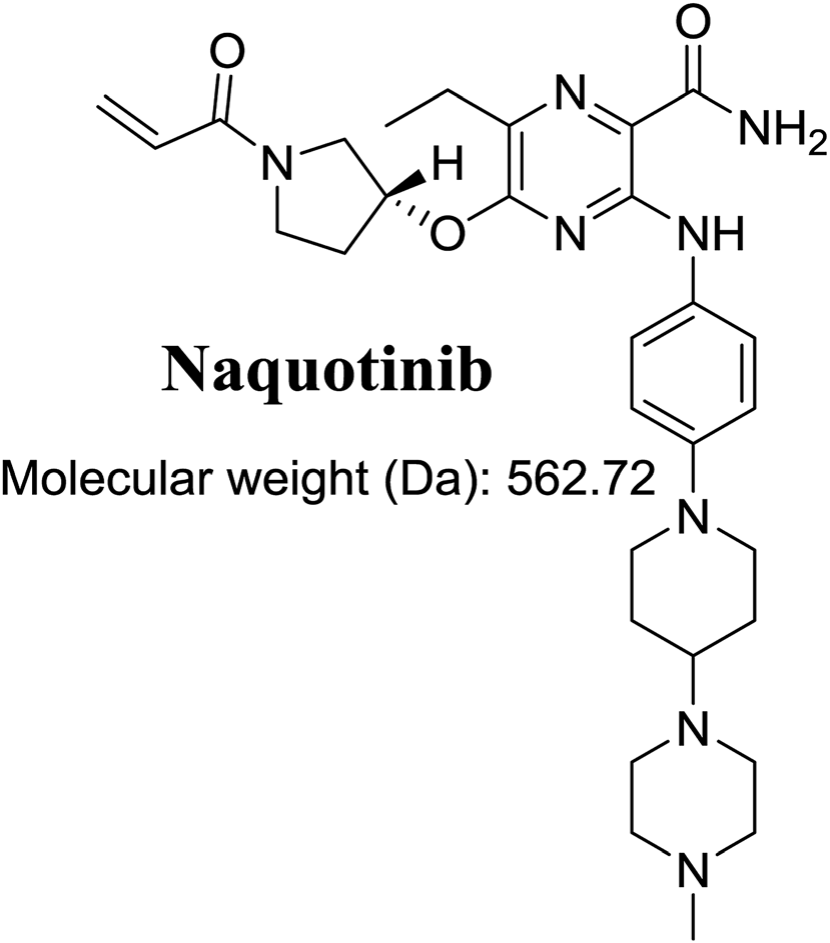
Chemical structure of naquotinib (NQT).

The International Union of Pure and Applied Chemistry name of NQT is 5-[(1-acryloyl-3-pyrrolidinyl)oxy]-6-ethyl-3-({4-[4-(4-methyl-1-piperazinyl)-1-piperidinyl]phenyl}amino)-2-pyrazinecarboxamide (Fig. 1). Its chemical structure includes cyclic tertiary amine rings (piperazine and pyrazine moieties) and an acryloylpyrrolidine group. Cyclic tertiary amine rings can undergo bioactivation by iminium ion generation.^24–27^ The acryloylpyrrolidine moiety can undergo oxidative dealkylation, forming aldehyde intermediates that can be captured using methoxyamine.^28^ Glutathione and its derivatives are highly nucleophilic and react poorly with strong electrophiles. However, the iminium ion and aldehydes are electrophiles that can be trapped using potassium cyanide and methoxyamine, respectively.^17,24,25^ The adducts formed by nucleophilic–electrophilic interactions are considered stable and can be separated and identified by LC-MS/MS.^22–24,29,30^ We hypothesize that these reactive intermediates are potentially involved in the reported side effects of NQT.

Accordingly, in this study, we used LC-MS/MS to screen for *in vitro* metabolites of NQT formed during incubation with human liver microsomes (HLMs) and then evaluated the generation of reactive electrophiles using capturing agents.

## 2. Chemicals and methods

### 2.1. Chemicals

All chemicals and solvents were of analytical grade. NQT was purchased from Med Chem Express (Princeton, NJ, USA). Acetonitrile (ACN), pooled human liver microsomes (HLMs, M0567), potassium cyanide and formic acid were procured from Sigma-Aldrich (St. Louis, MO, USA). HPLC-grade water (H_2_O) was obtained from the Milli-Q plus filtration system (Millipore, Billerica, MA, USA).

### 2.2. Chromatographic conditions

Separation and characterization of *in vitro* NQT metabolites and reactive intermediates from the HLM-incubation mixtures was done utilizing an Agilent Triple Quadrupole system consisting of an Agilent 1200 LC as an HPLC systems and an Agilent 6410 QqQ as a mass detector (Agilent Technologies, Palo Alto, CA, USA) with electrospray ionization (ESI) source. Separation of the components of the metabolic mixtures was performed on a C_18_ column (length, 150 mm; internal diameter, 2.1 mm; and particle size, 3.5 μm). The column temperature was adjusted at 23 ± 2 °C, and we used a gradient mobile phase at a flow rate of 0.2 mL min^−1^ and consisting of 10 mM ammonium formate in H_2_O (solvent A; pH 4.2) and ACN (solvent B). The stepwise-gradient system involved solvent B (5%; 0–5 min), solvent B (5–60%; 5–50 min), solvent B (60–80%; 50–70 min), and solvent B (80–5%; 70–75 min), with a post time of 15 min. The sample injection volume was 5 μL and had a total run time of 75 min, with the mass parameters optimized for vandetanib. Fragmentation of NQT, phase I metabolites, and related adducts were performed in the collision cell by collision-induced dissociation (CID). Detection was performed on a mass detector operated using a positive ESI source.^14,15^ Drying gas (N_2_) was used as at a flow rate of 12 L min^−1^, and collision gas (high-purity N_2_) was used at a pressure of 60 psi. Source temperature, capillary voltage, fragmentor voltage, and collision energy were set to 350 °C, 4000 V, 135 V, and 22 eV, respectively. Mass Hunter software (Agilent Technologies) was used to for instrument management and data acquisition.

### 2.3. HLM incubation

The screen for *in vitro* metabolites of NQT was performed by incubating 30 μM NQT with 1.0 mg mL^−1^ HLMs in phosphate buffer (50 mM, pH 7.4) and 3.3 mM MgCl_2_. The incubation time and temperature were 2 h and 37 °C, respectively. The metabolic incubation reactions were performed in a shaking water bath. Initiation of the NQT metabolic reaction was performed by adding NADPH (1.0 mM) and quenched by the addition of 2 mL ice-cold acetonitrile, which was also used as a protein precipitating agent. Precipitates were removed by centrifugation at 9000 × *g* (15 min, 4 °C), and the supernatants were transferred to clean tubes and evaporated to dryness. Residues were reconstituted in mobile phase solvent. Ten microliters of each reconstituted sample was injected and analyzed using LC-MS/MS.^35,36^

### 2.4. Identification of NQT reactive metabolites

After full MS scanning over a selected range, extracted ion chromatograms of targeted *m*/*z* peaks were utilized to locate expected *in vitro* metabolites in the total ion chromatograms. Fragmentation suspected peaks using product ion (PI) mode were used to confirm the metabolite chemical structure by reconstructing daughter ions (DIs). The fragmentation pattern (FP) of NQT was used as a lead for interpretation and confirmation of the proposed chemical structures of *in vitro* metabolites and reactive intermediates generated in the metabolism of NQT.

The same HLM incubation with NQT was performed in the presence of the capturing agent (methoxyamine or potassium cyanide) to trap bioactive intermediates. To confirm the outcomes, all experiments were repeated three times, with appropriate controls.

## 3. Results and discussion

### 3.1. Fragmentation analysis of NQT

**Fig. 2:**
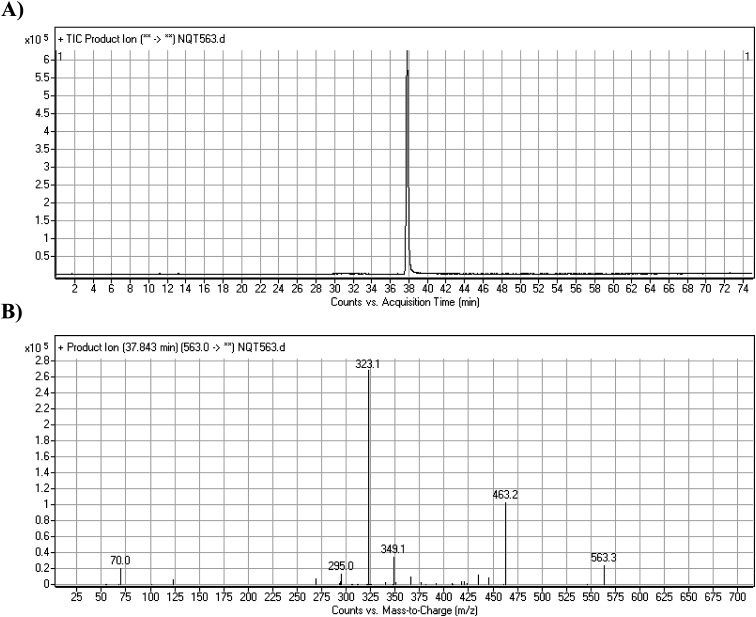
PI chromatogram at *m*/*z* 563 showing the NQT peak at 37.8 min (A). PI mass spectrum of NQT (B).

**Scheme 1:**
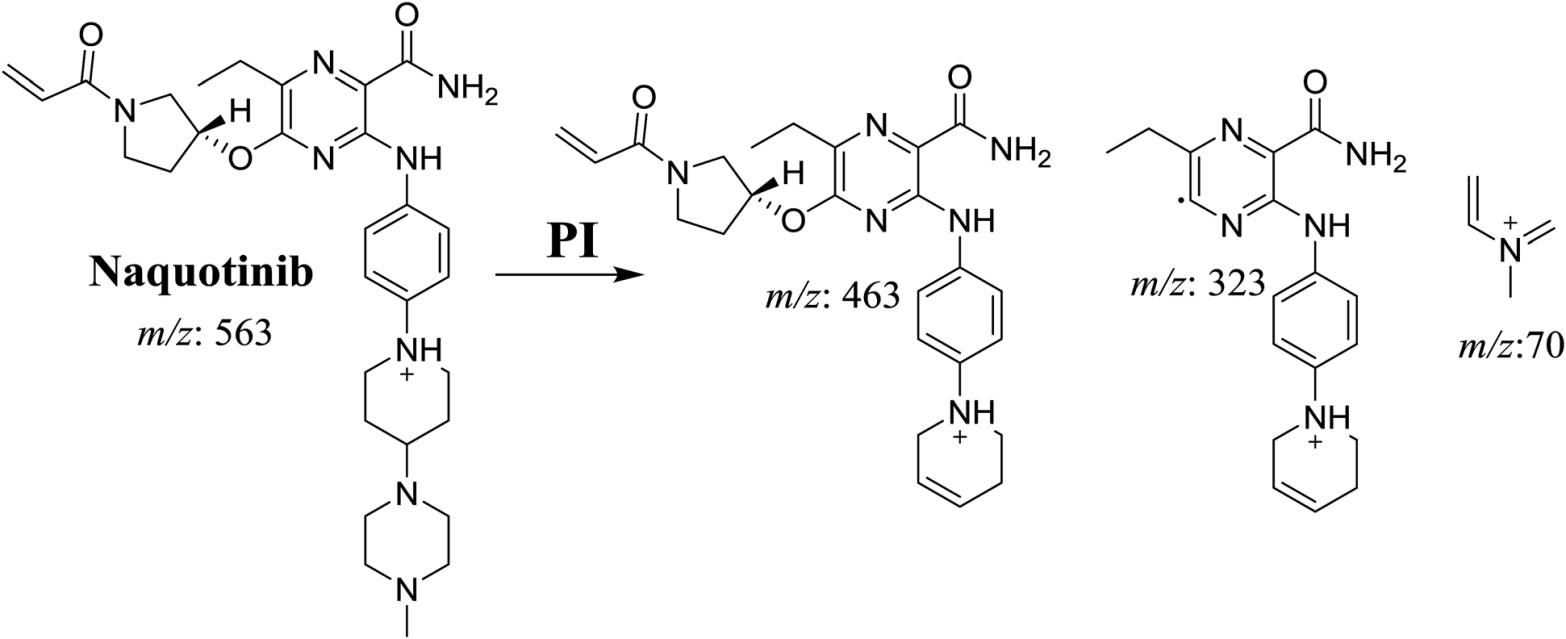
Structural formulas of NQT and corresponding MS/MS fragments.

The NQT molecular ion peak (MIP) was found at 37.8 min in the daughter ion chromatogram (DIC; Fig. 2A). Fragmentation of the PI at *m*/*z* 563 generated many DIs. Three DIs at *m*/*z* 463, 323, and 70 represented qualitative soft points inside the NQT structure (Fig. 2B, Scheme 1).

### 3.2. Identification of NQT *in vitro* metabolites and reactive intermediates

Four phase I metabolic reactions produced eight metabolites by *N*-demethylation, oxidation, hydroxylation, and reduction. One cyano and two methoxyamine adducts were identified (Table 1).

**Table 1:** Phase I and reactive metabolites of NQT

	MS scan	Most abundant fragment ions	Rt. (min)	Metabolic reaction
NQT	563	463, 323, 70	37.8	Main drug

**Phase I metabolites**				
NQT549	549	463, 435, 323	36.9	*N*-Demethylation in the piperazine ring
NQT565	565	465, 437, 324, 70	31.2	Reduction metabolic reaction in amide group
NQT577a	577	477, 323, 101	41.1	α-Oxidation in pyrrolidine ring
NQT577b	577	477, 337, 70	41.4	α-Oxidation in piperidine ring
NQT577c	577	463, 323, 70	42.4	α-Oxidation in piperazine ring
NQT579a	579	479, 323	38.0	Hydroxylation in pyrrolidine ring
NQT579b	579	463, 323, 70	38.4	Hydroxylation in piperazine ring
NQT579c	579	479, 339, 321, 70	41.6	Hydroxylation in piperidine ring

**Reactive metabolites**				
NQT588	588	561, 461, 321	33.8	Cyano conjugation in piperidine ring
NQT566	566	466, 324, 125, 70	32.7	Oxidative dealkylation of acryl group then methoxyamine oximer formation
NQT439	580	480, 338, 125	41.2	α-Oxidation in piperidine ring and oxidative dealkylation of acryl group then methoxyamine oximer formation

#### 3.2.1. Identification of the NQT549 phase I metabolite

**Fig. 3:**
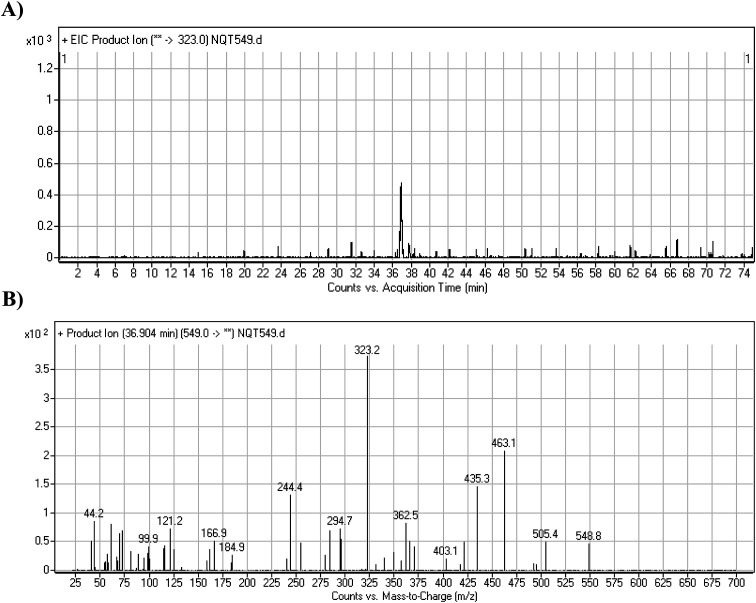
PI chromatogram of the NQT549 peak at 36.9 min (A). PI mass spectrum of NQT549 (B).

**Scheme 2:**
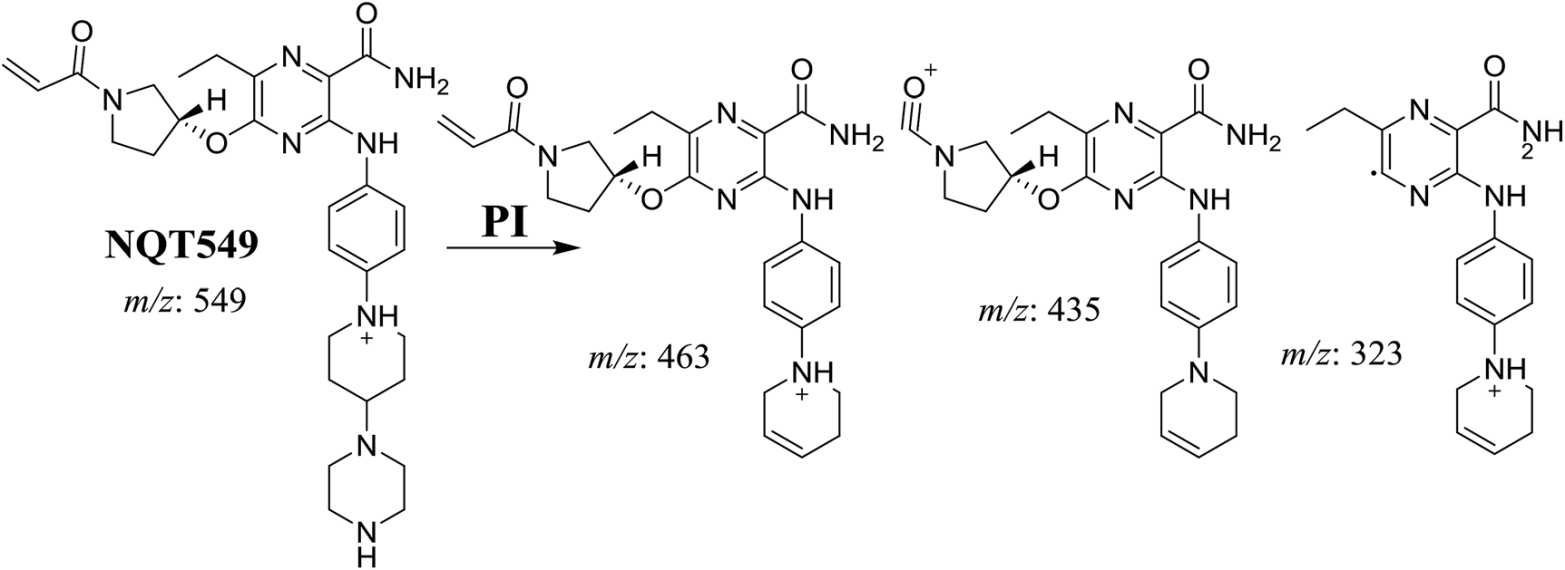
Structural formulas of NQT549 and corresponding MS/MS fragments.

The NQT549 MIP was found at 36.9 min in the DIC (Fig. 3A). Fragmentation of the PI at *m*/*z* 549 generated three DIs at *m*/*z* 463, 435, and 323. Compared with the FP of NQT, DIs at *m*/*z* 463 and 323 revealed that the *N*-demethylation metabolic reaction occurred in the *N*-methyl piperazine ring, which was consistent with the absence of the DI at *m*/*z* 70 (Fig. 3B, Scheme 2).

#### 3.2.2. Identification of the NQT465 phase I metabolite

**Fig. 4:**
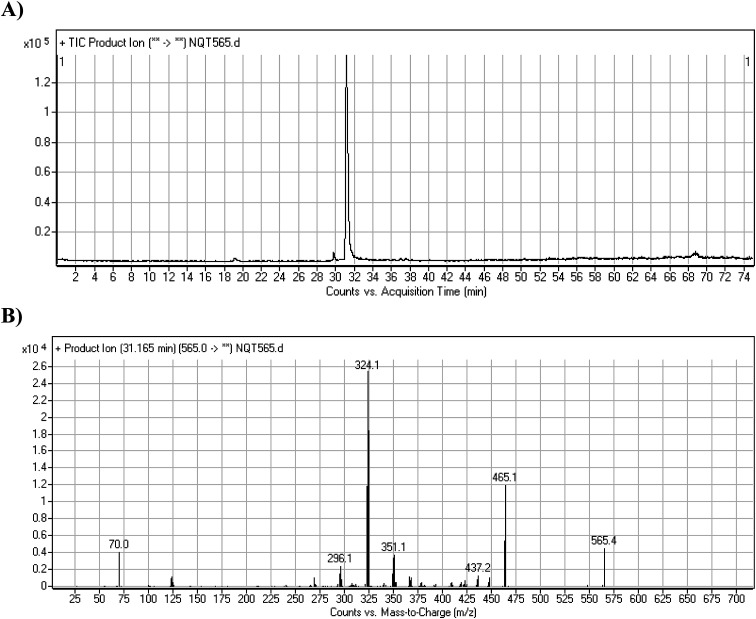
PI chromatogram of the NQT565 peak at 31.2 min (A). PI mass spectrum of NQT565 (B).

**Scheme 3:**
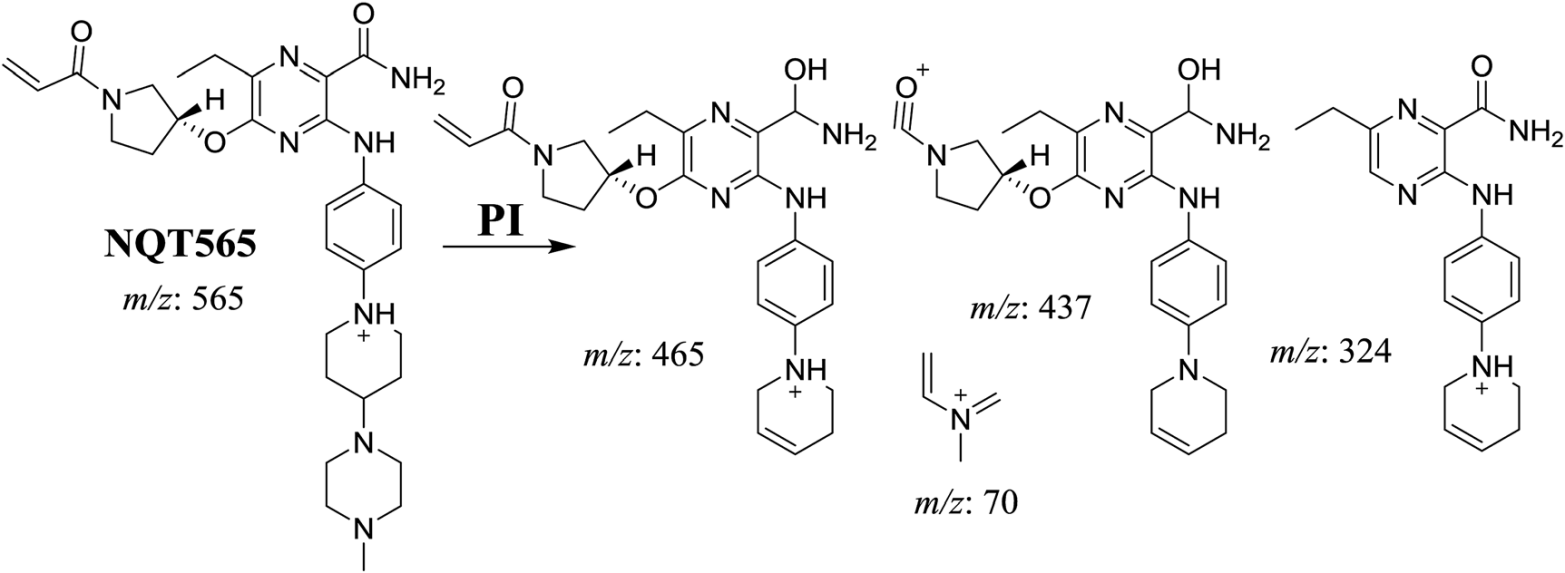
Structural formulas of NQT565 and corresponding MS/MS fragments.

The NQT565 MIP was found at 31.2 min in the DIC (Fig. 4A). Fragmentation of the PI at *m*/*z* 465 generated four DIs at *m*/*z* 465, 437, 324, and 70. Compared with the FP of NQT, DIs at *m*/*z* 465, 437, and 324 revealed that the reduction metabolic reaction occurred in the amide group, consistent with the other DI at *m*/*z* 70 (Fig. 4B, Scheme 3).

#### 3.2.3. Identification of NQT577 phase I metabolites

**Fig. 5:**
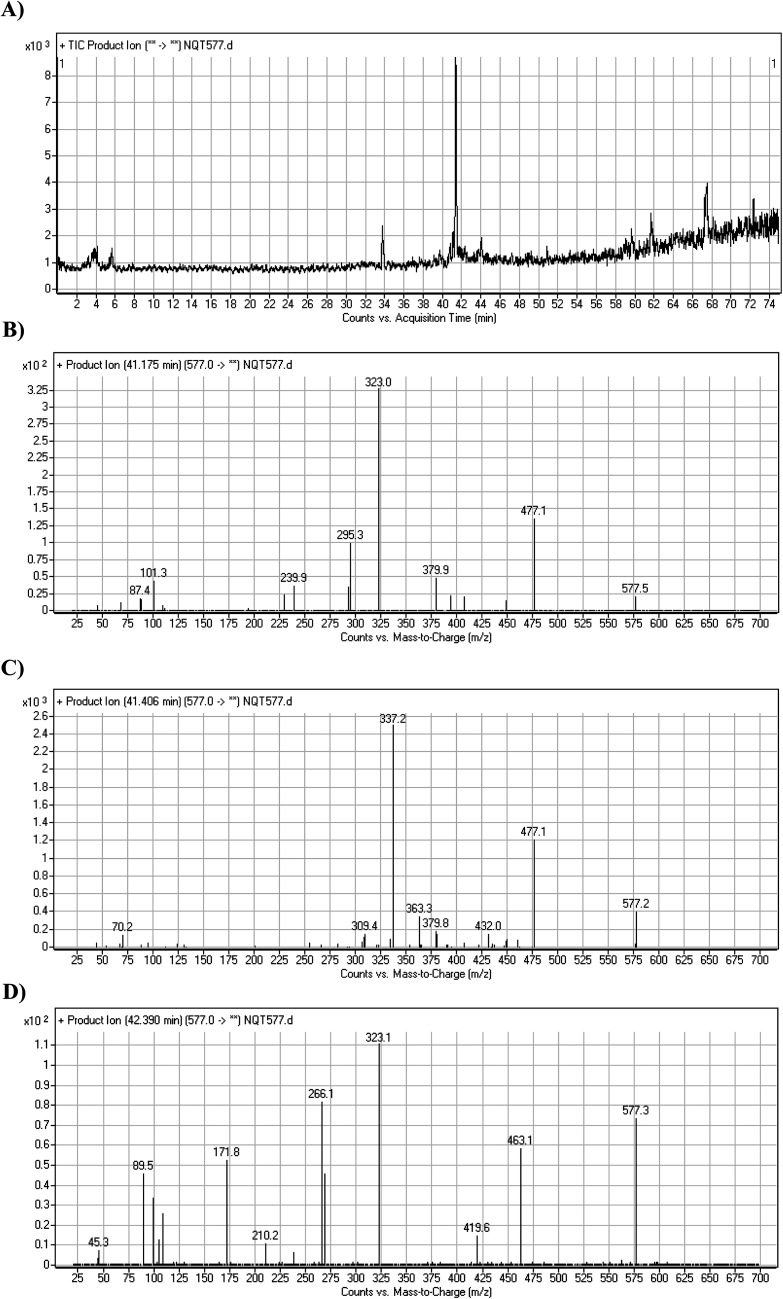
PI chromatogram of NQT577a, NQT577b, and NQT577c peaks at 41.1, 41.4, and 42.4 min, respectively (A). PI mass spectra of NQT577a (B), NQT577b (C), and NQT577c (D).

NQT577a, NQT577b, and NQT577c MIP peaks were found at 41.1, 41.4, and 42.4 min, respectively, in the DIC (Fig. 5A). Fragmentation of the MIP at *m*/*z* 577 generated different DIs (Fig. 5B–D).

**Scheme 4:**
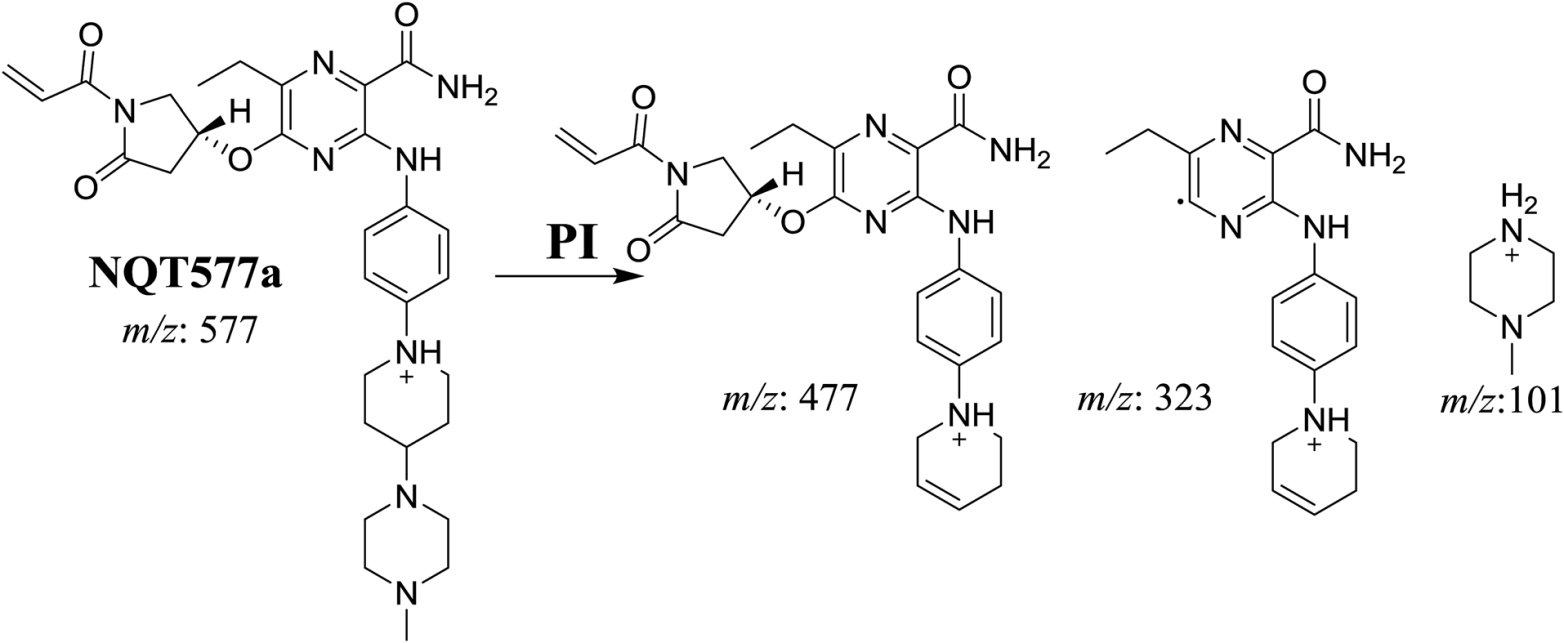
Structural formulas of NQT577a and corresponding MS/MS fragments.

For NQT577a, fragmentation of the MIP at *m*/*z* 577 generated three DIs at *m*/*z* 477, 323, and 101. Compared with the FP of NQT, DIs at *m*/*z* 477 and 323 revealed that no metabolic reaction occurred for either the piperazine or piperidine groups. The oxidation metabolic reaction was proposed to occur in the pyrrolidine group (Scheme 4).

**Scheme 5:**
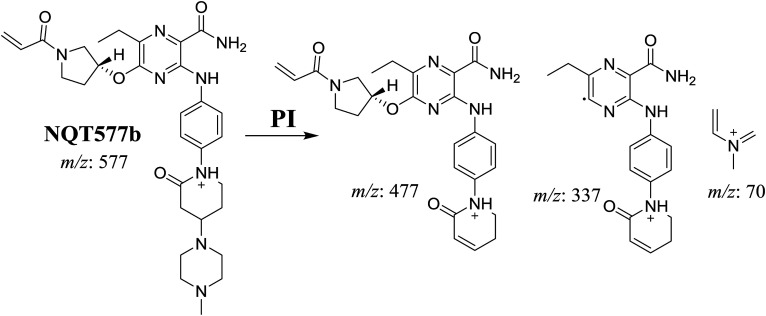
Structural formulas of NQT577b and corresponding MS/MS fragments.

For NQT577b, fragmentation of the MIP at *m*/*z* 577 generated three DIs at *m*/*z* 477, 337, and 101. Compared with the FP of NQT, DIs at *m*/*z* 477 revealed that no metabolic reaction occurred in the piperazine ring. DIs at *m*/*z* 337 revealed that the oxidation metabolic reaction occurred in the piperidine ring, consistent with the other DI at *m*/*z* 70 (Scheme 5).

**Scheme 6:**
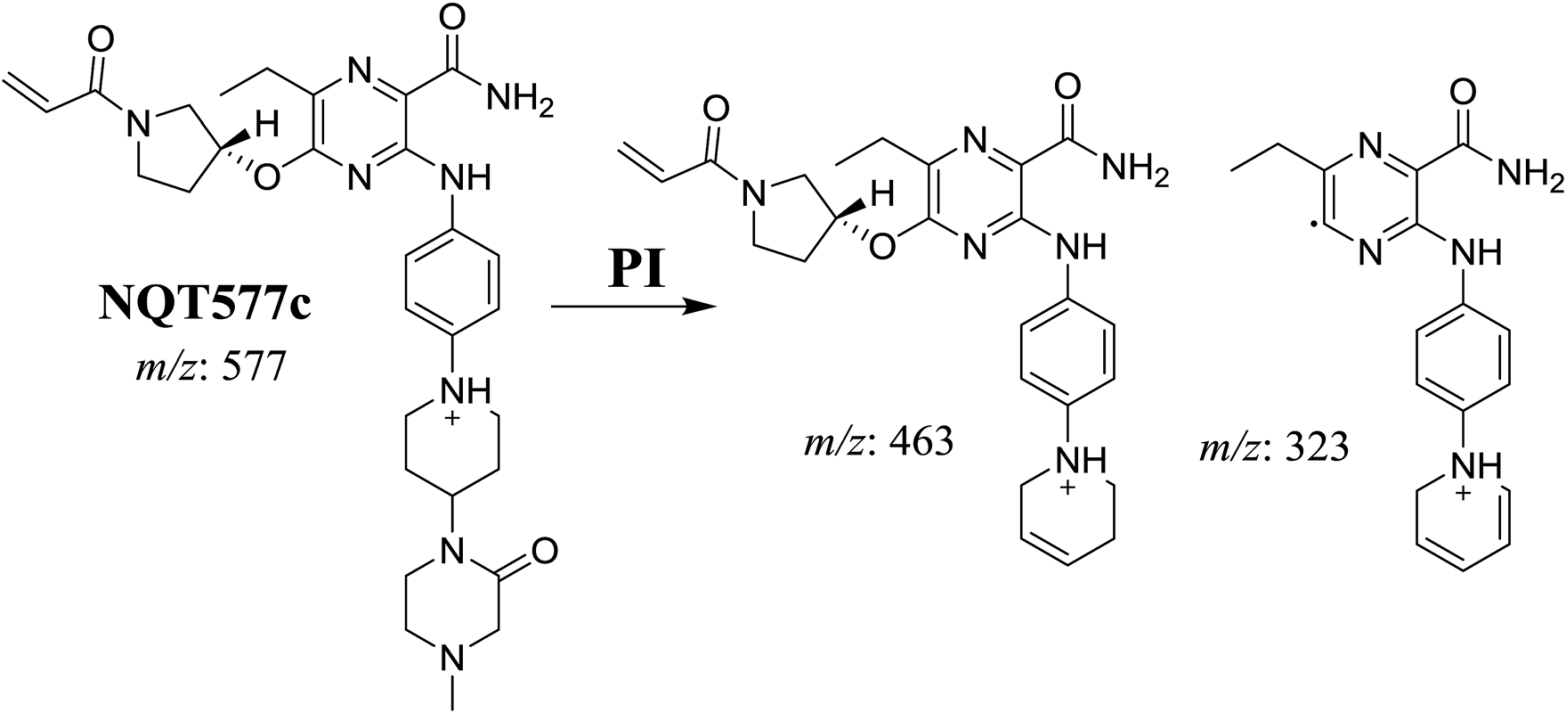
Structural formulas of NQT577c and corresponding MS/MS fragments.

For NQT577c, fragmentation of the MIP at *m*/*z* 577 generated three DIs at *m*/*z* 463 and 323. Compared with the FP of NQT, DIs at *m*/*z* 463 revealed that no metabolic reaction occurred in the pyrrolidine ring. DIs at *m*/*z* 323 revealed that no metabolic reaction occurred in the piperidine ring. The oxidation metabolic reaction was proposed to occur in the piperazine group (Scheme 6).

#### 3.2.4. Identification of NQT579 phase I metabolites

**Fig. 6:**
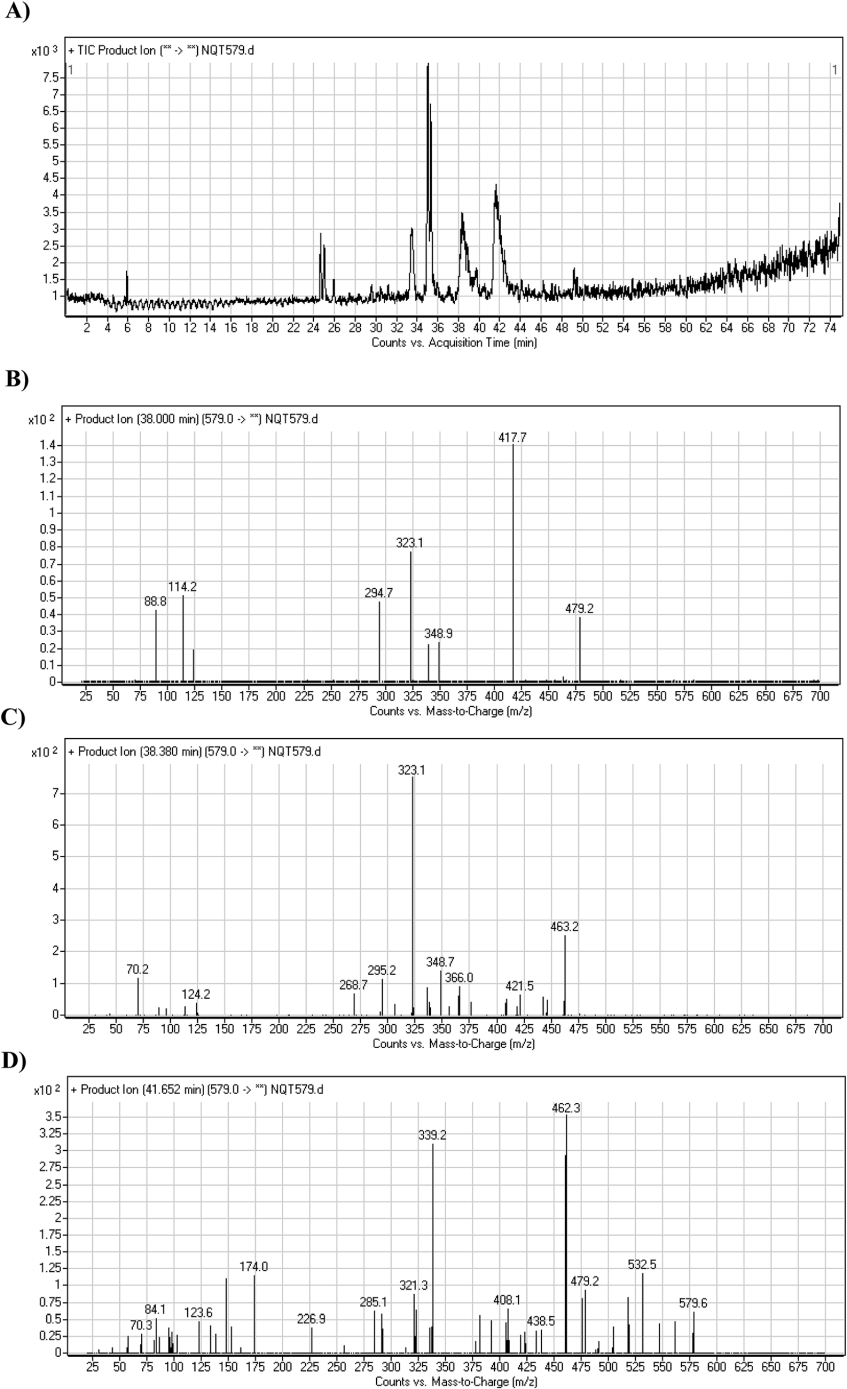
PI chromatogram of NQT579a, NQT579b, and NQT579c peaks at 38.0, 38.4, and 41.6 min, respectively (A). PI mass spectra of NQT579a (B), NQT579b (C), and NQT579c (D).

NQT579a, NQT579b, and NQT579c MIP peaks were found at 38.0, 38.4, and 41.6 min, respectively, in the DIC (Fig. 6A). Fragmentation of the MIP at *m*/*z* 579 generated different DIs (Fig. 6B–D).

**Scheme 7:**
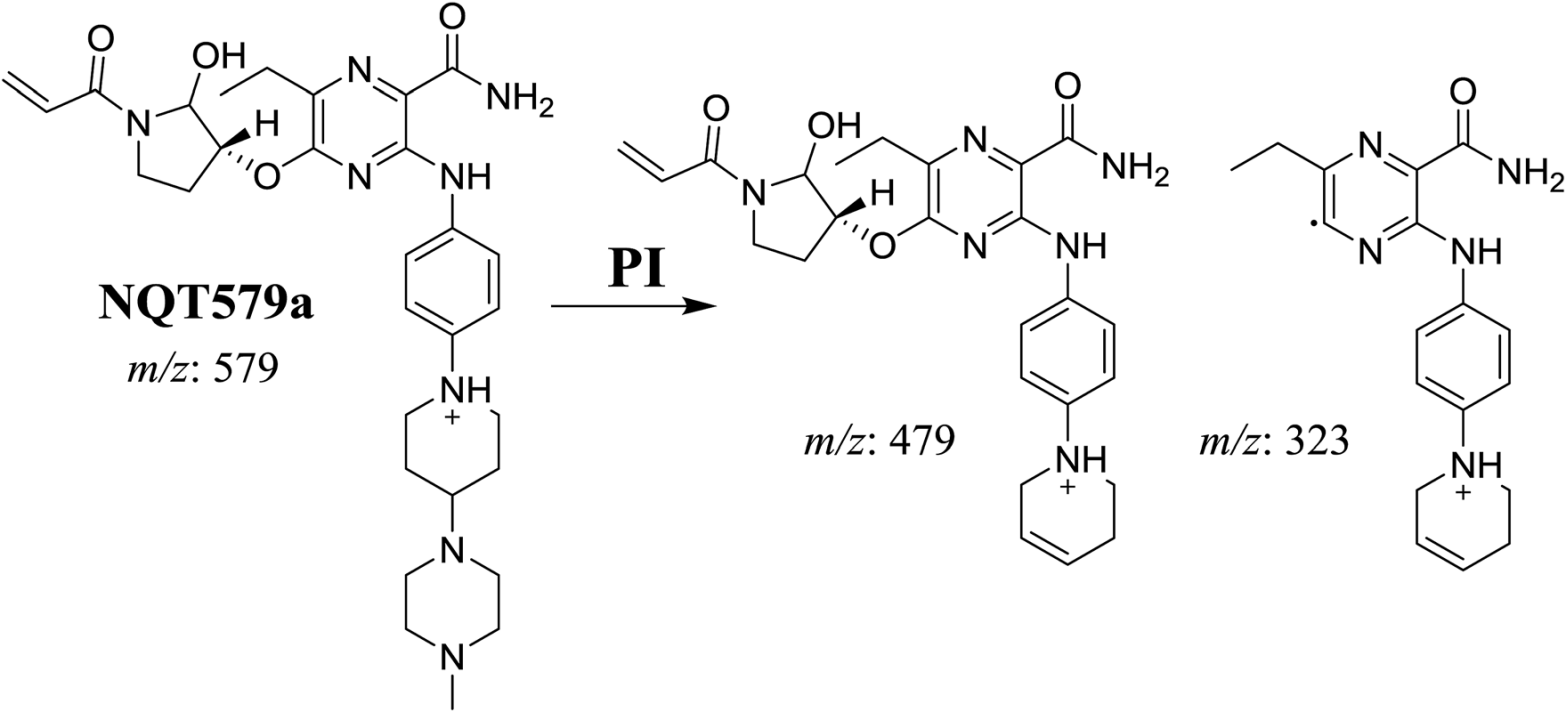
Structural formulas of NQT579a and corresponding MS/MS fragments.

For NQT579a, fragmentation of the MIP at *m*/*z* 579 generated two DIs at *m*/*z* 479 and 323. Compared with the FP of NQT, DIs at *m*/*z* 479 and 323 revealed that no metabolic reaction occurred for either the piperazine or piperidine groups. The hydroxylation metabolic reaction was proposed to occur in the pyrrolidine group (Scheme 7).

**Scheme 8:**
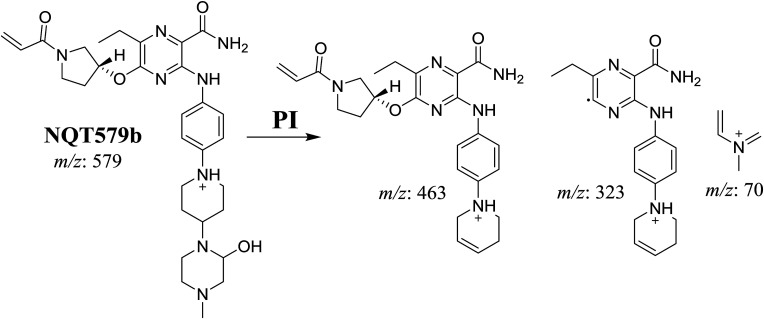
Structural formulas of NQT579b and corresponding MS/MS fragments.

For NQT579b, fragmentation of the MIP at *m*/*z* 579 generated three DIs at *m*/*z* 463, 323, and 70. Compared with the FP of NQT, DIs at *m*/*z* 463 revealed that no metabolic reaction occurred in the pyrrolidine ring, and DIs at *m*/*z* 323 revealed that no metabolic reaction occurred in piperidine ring. The hydroxylation metabolic reaction was proposed to occur in the piperazine group (Scheme 8).

**Scheme 9:**
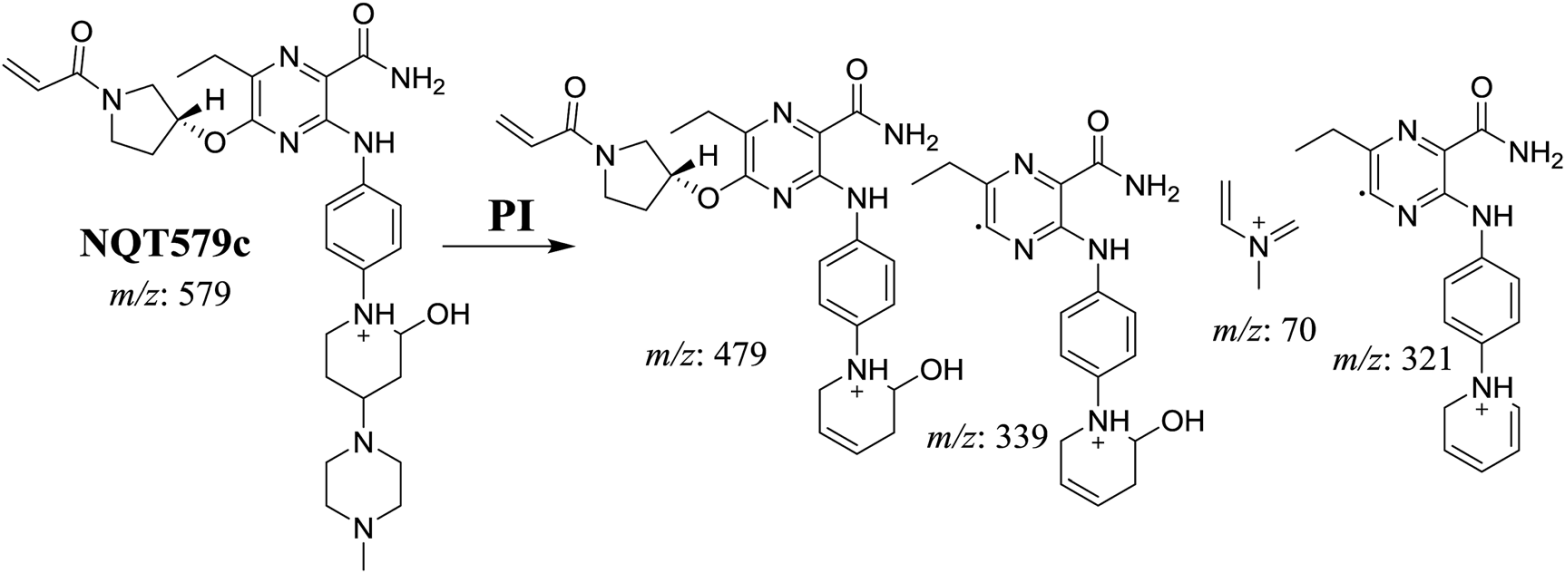
Structural formulas of NQT579c and corresponding MS/MS fragments.

For NQT579c, fragmentation of the MIP at *m*/*z* 579 generated four DIs at *m*/*z* 479, 339, 321, and *m*/*z* 70. Compared with the FP of NQT, DIs at *m*/*z* 479 revealed that no metabolic reaction occurred in the piperazine ring, and DIs at *m*/*z* 339 revealed that the hydroxylation metabolic reaction occurred in the piperidine ring, consistent with the other DIs at *m*/*z* 321 and 70 (Scheme 9).

### 3.3. Reactive metabolites

One cyano and two methoxyl adducts were identified after incubation of NQT with HLMs in the presence of trapping agents.

#### 3.3.1. Identification of NQT588 cyano conjugate

**Fig. 7:**
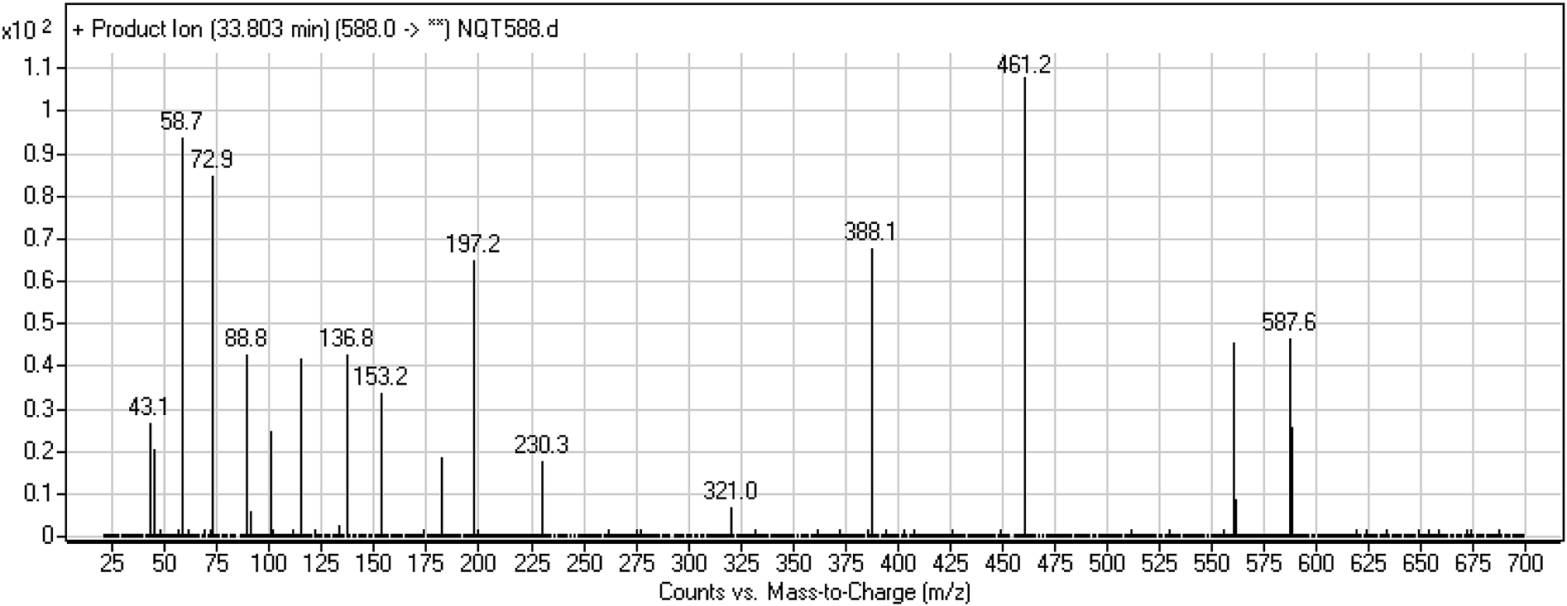
PI mass spectrum of NQT588.

**Scheme 10:**
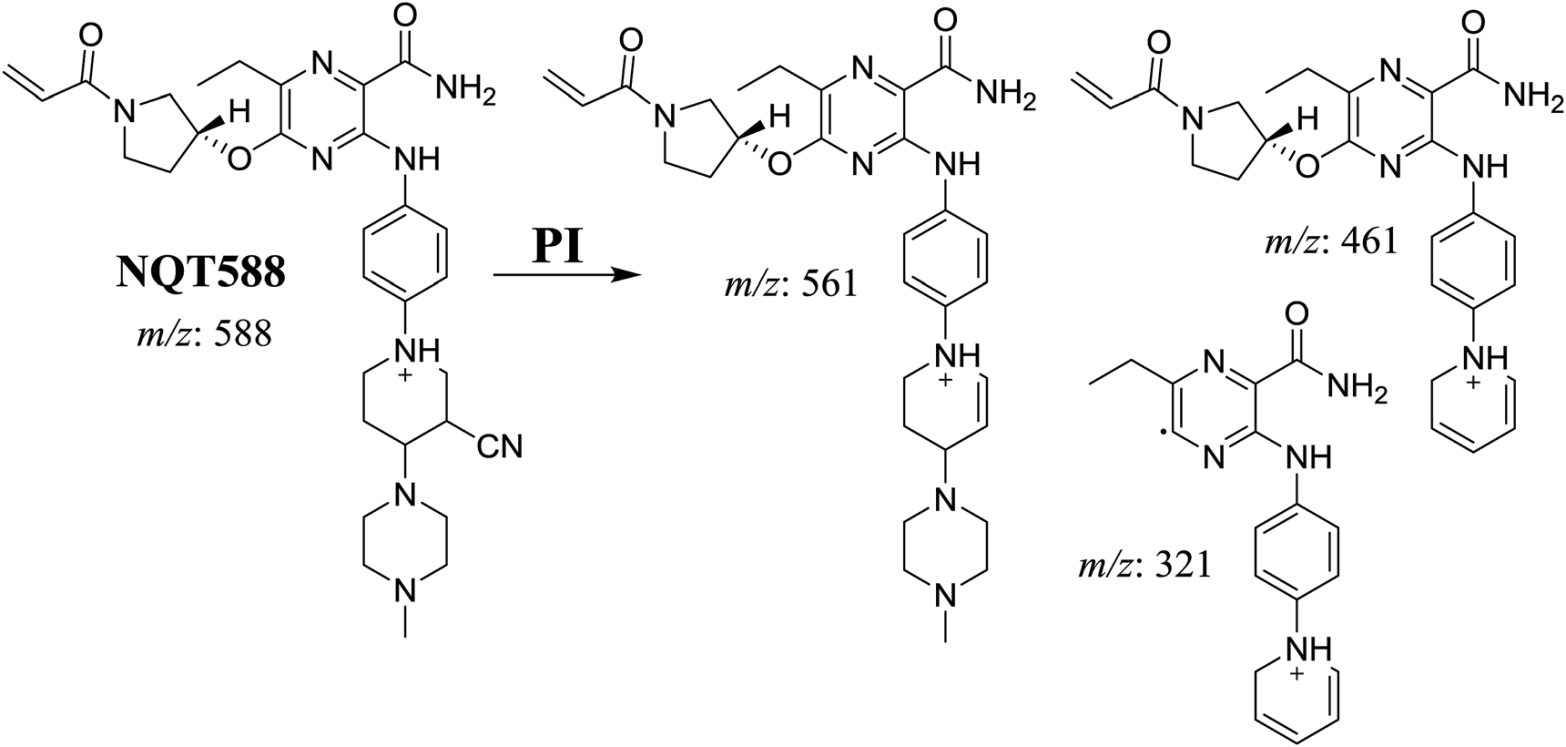
Structural formulas of NQT588 and corresponding MS/MS fragments.

The NQT588 MIP was found at 33.8 min in the DIC (Fig. 7). Fragmentation of the PI at *m*/*z* 588 generated four DIs at *m*/*z* 561, 461, and 321. The DI at *m*/*z* 461 indicated the loss of HCN, which is characteristic of cyano conjugates. Compared with the FP of NQT, DIs at *m*/*z* 461 and 321 revealed that the metabolic bioactivation reaction and cyano conjugation occurred in the piperidine ring (Scheme 10).

#### 3.3.2. Identification of the NQT566 methoxyamine conjugate

**Fig. 8:**
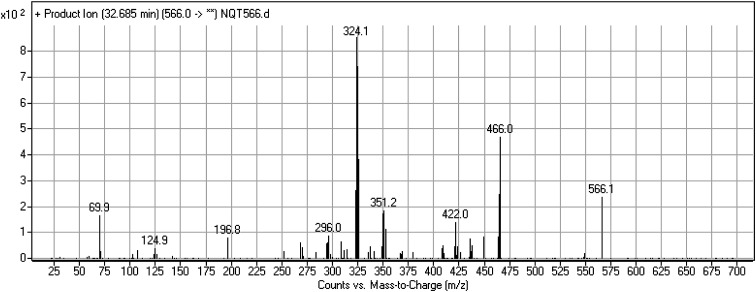
PI mass spectrum of NQT566.

**Scheme 11:**
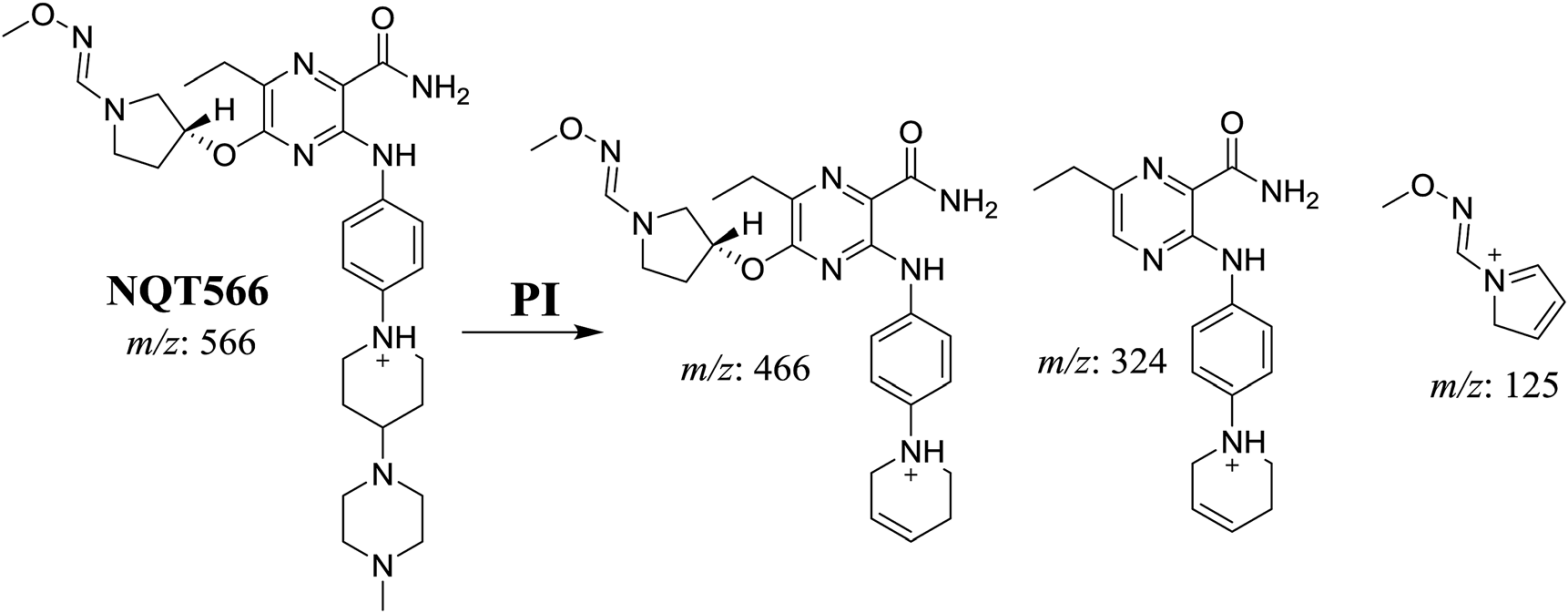
Structural formulas of NQT566 and corresponding MS/MS fragments.

The NQT566 MIP was found at 32.7 min in the DIC (Fig. 8). Fragmentation of the PI at *m*/*z* 466 generated four DIs at *m*/*z* 466, 324, 125, and 70. Compared with the FP of NQT, DIs at *m*/*z* 466 and 125 revealed oxime formation, which was consistent with the other DI at *m*/*z* 70. Oxidative dealkylation of the acryl group occurred from the acryloylpyrrolidine moiety, forming an aldehyde intermediate that generated an oxime in the presence of the methoxyl amine (Scheme 11).

#### 3.3.3. Identification of the NQT580 methoxyamine conjugate

**Fig. 9:**
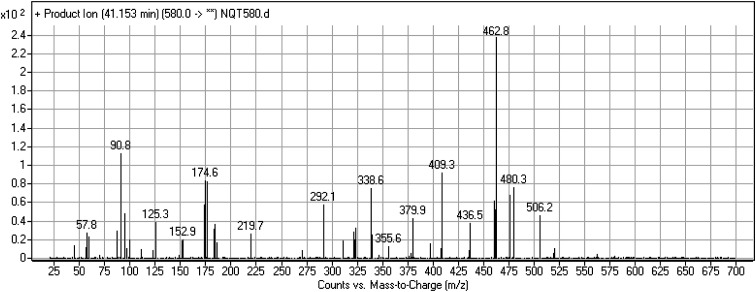
PI mass spectrum of NQT580.

**Scheme 12:**
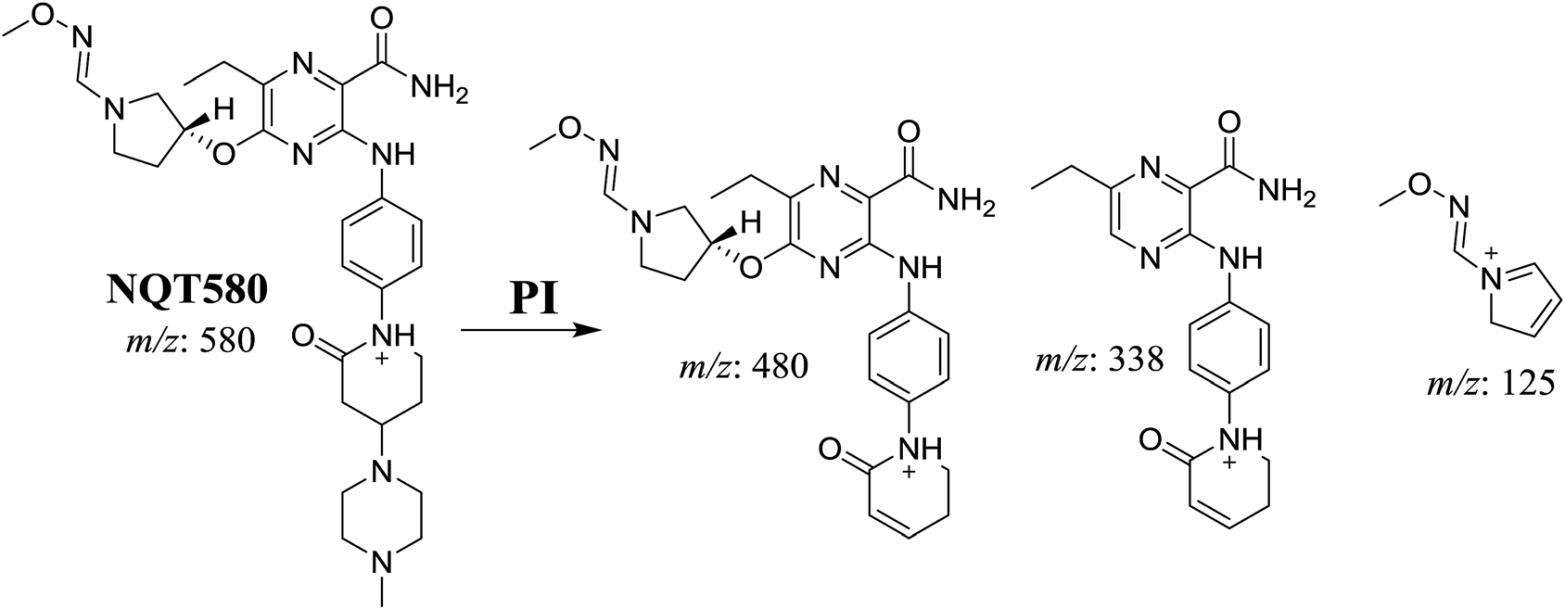
Structural formulas of NQT580 and corresponding MS/MS fragments.

The NQT580 MIP was found at 41.2 min in the DIC (Fig. 9). Fragmentation of the PI at *m*/*z* 580 generated four DIs at *m*/*z* 480, 338, and 125. Compared with the FP of NQT, DIs at *m*/*z* 480 and 125 revealed oxime formation and α-oxidation of the piperidine ring, consistent with the other DI at *m*/*z* 338. Oxidative dealkylation of the acryl group occurred at the acryloylpyrrolidine moiety, forming an aldehyde intermediate that generated an oxime in the presence of methoxyl amine (Scheme 12).

### 3.4. Mechanism of NQT bioactivation

**Scheme 13:**
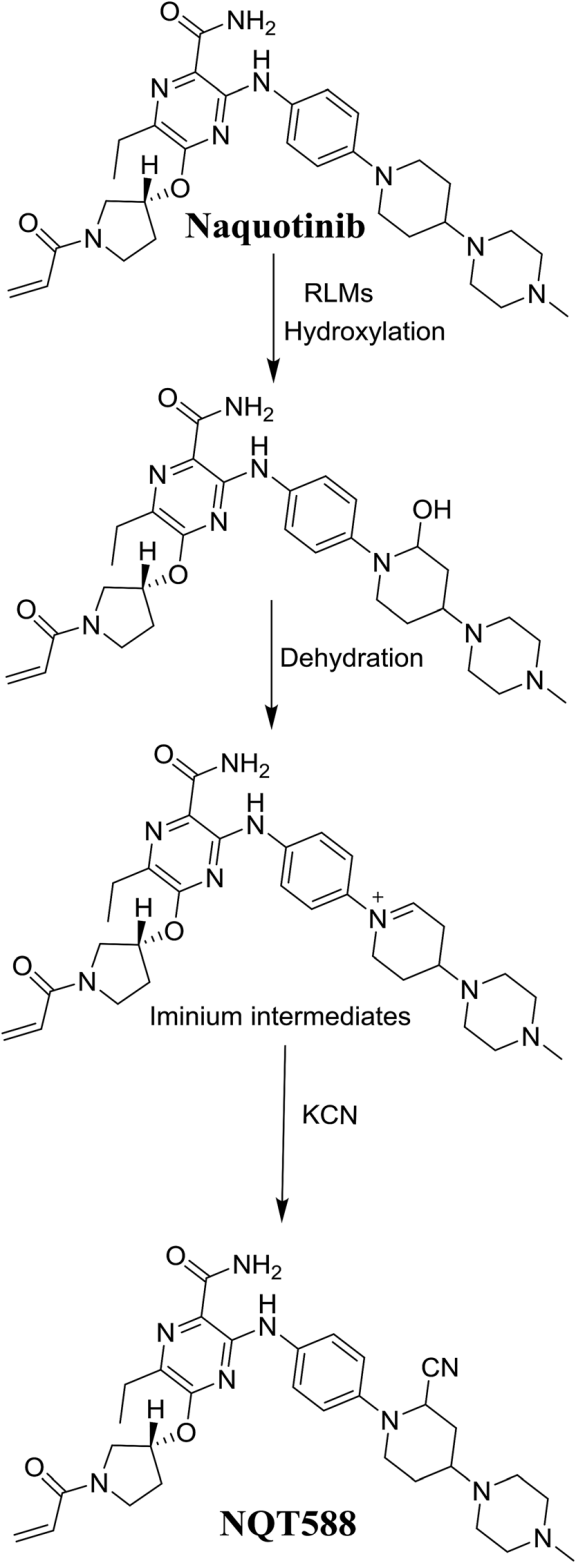
Proposed mechanism of iminium intermediate formation during NQT metabolism and a potential trapping strategy.

The production of the NQT588 cyanide adduct confirmed the generation of iminium intermediates. The hydroxylation metabolic reaction of the piperidine ring in NQT followed by one water molecule loss created iminium electrophiles that were unstable but could be captured by a cyanide nucleophile to form a stable adduct (Scheme 13). The mechanism of the iminium intermediate formation and NQT bioactivation has been previously studied on cyclic tertiary amine-containing drugs.^28,31–33^

**Scheme 14:**
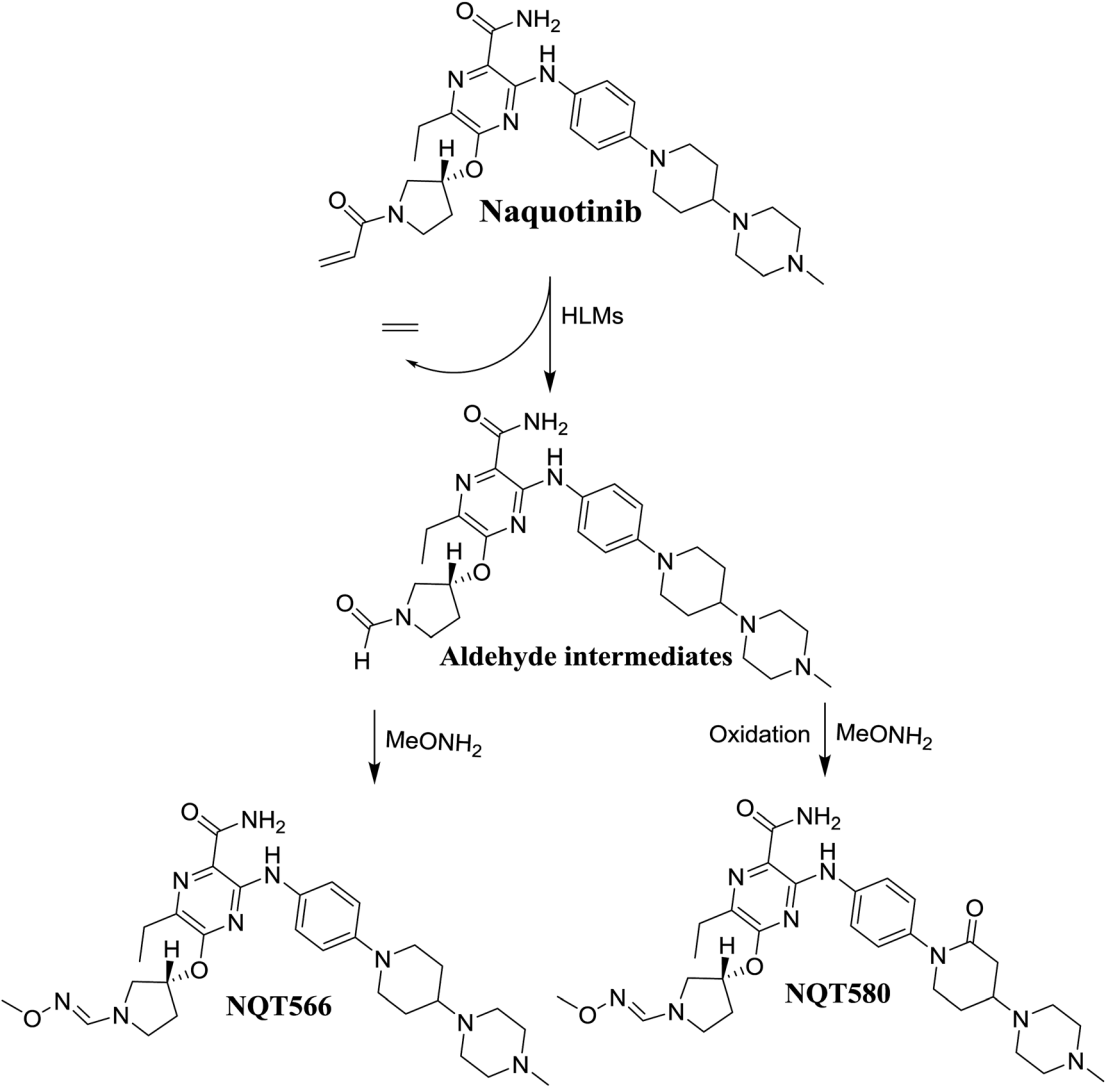
Proposed mechanism of aldehyde generation.

The formation of NQT566 and NQT580 demonstrated the generation of aldehyde intermediates in the NQT metabolism. The aldehyde electrophiles were formed by oxidative dealkylation and captured with methoxyamine, forming oxime (NQT566 and NQT580). Oxidative dealkylation of the acryloylpyrrolidine group formed an aldehyde that was captured by methoxyamine, forming NQT566. Both oximes were stable and identified using LC-MS/MS (Scheme 14). Aldehyde formation of acryloyl group-containing drugs has been discussed previously.^34,35^

## 4. Conclusions

**Fig. 10:**
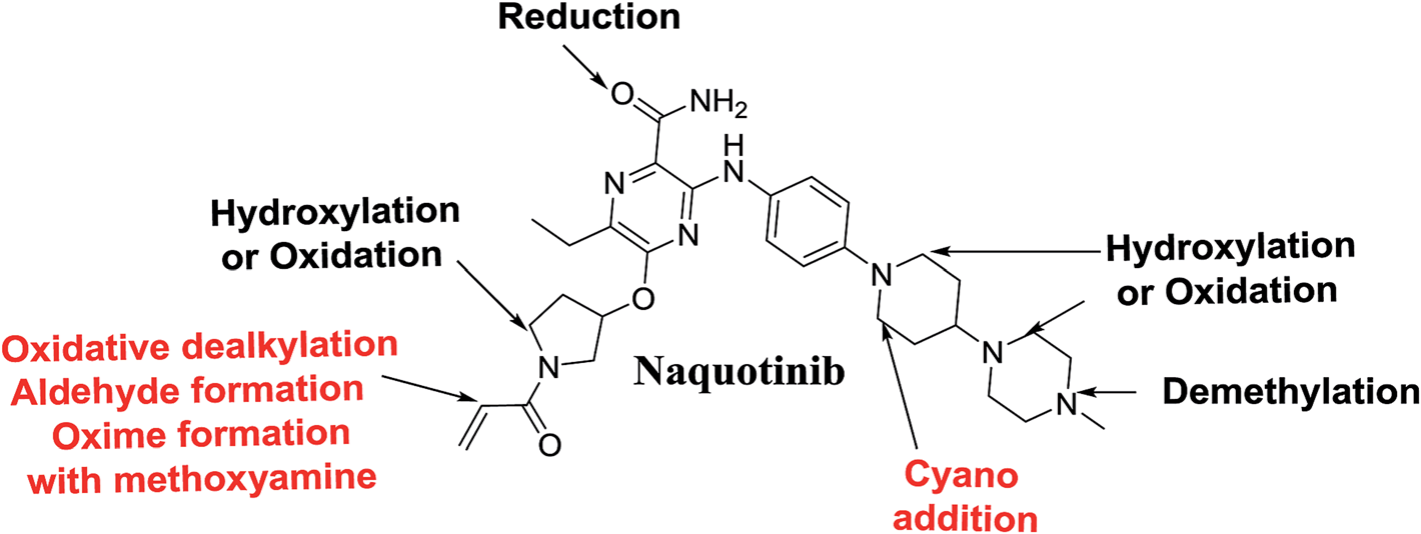
NQT chemical structure showing different metabolic phase I reactions and bioactive centers.

*N*-Demethylation, oxidation, hydroxylation, and reduction metabolic reaction generated eight phase I NQT metabolites. Oxidative dealkylation of the acryloylpyrrolidine group formed aldehydes that were captured by MeONH_2_, forming NQT566 and NQT580. Hydroxylation of the piperidine ring in NQT, followed by dehydration created an iminium electrophile that was captured by KCN, forming NQT588. Bioactivation resulted in the formation of one cyano adduct and two methoxyamine adducts of NQT (Fig. 10). These findings established a basis for further work on NQT toxicity.

## Conflicts of interest

The authors declare no conflicts of interest.

## Supplementary Material
